# The potential role of the KFG and KITLG proteins in preventing granulosa cell apoptosis in *Bubalus bubalis*

**DOI:** 10.1186/s43141-023-00480-2

**Published:** 2023-03-31

**Authors:** Deepak Panwar, Leena Rawal, Sher Ali

**Affiliations:** 1 Molecular Genetics Laboratory, National Institute of Immunology, Aruna Asaf Ali Marg, New Delhi, 110067 India; 2 Present Address: National Reference Laboratory, Dr. Lal Pathlabs, B7 Road, Block E, Rohini Sector 18, New Delhi, 110085 India; 3 Present Address: Department of Personalized Medicine, VC Office, Era University, Lucknow, 226003 India

**Keywords:** Apoptosis, Granulosa cells, Real-time PCR, KGF, KITLG

## Abstract

**Background:**

The dynamics of mammalian follicular development and atresia is an intricate process involving the cell-cell communication mediated by secreted ovarian factors. These interactions are critical for oocyte development and regulation of follicular atresia which in part are mediated by keratinocyte growth factor (KGF) and kit ligand (KITLG), but their roles in the regulation of apoptosis in buffalo granulosa cells have not yet been defined. During mammalian follicular development, granulosa cell apoptosis triggers the atresia so ~ 1% follicles reach the ovulation stage. In the present study, we used buffalo granulosa cells to examine the effects of KGF and KITLG in apoptosis regulation and investigated potential mechanism on Fas-FasL and Bcl-2 signaling pathways.

**Result:**

Isolated buffalo granulosa cells were cultured with KGF and KITLG proteins using different doses (0, 10, 20, and 50 ng/ml) independently or in combination. Expression analysis for both anti-apoptotic (*Bcl-2*, *Bcl-xL*, and *cFLIP*) and pro-apoptotic (*Bax, Fas,* and *FasL*) genes at transcriptional levels were carried out by real-time PCR. Upon treatments, expression levels of anti-apoptotic genes were significantly upregulated in a dose-dependent manner, showing an upregulation at 50 ng/ml (independently), and at 10 ng/ml in combination. Additionally, upregulation of growth-promoting factors, *bFGF*, and *α-Inhibin* was also observed.

**Conclusions:**

Our findings suggest the potential roles of KGF and KITLG in determining granulosa cell growth and regulating apoptosis.

## Background

Ovarian folliculogenesis is a dynamic and coordinated process involving follicle selection, follicular growth and dominance, ovulation, and formation of corpus luteum. Within the ovarian follicle, an oocyte is surrounded by epithelial-derived granulosa cells and mesenchymal-derived thecal cells. The fate of an individual follicle strongly depends on the stages of folliculogenesis and an effective bidirectional communication between the oocyte and the granulosa/theca cells. During folliculogenesis, many follicles are recruited but only a few develop and become dominant to reach the preovulatory stage for eventual ovulation [1, 2]. The remaining follicles undergo a degenerating process known as atresia. As a result, more than 99% of all follicles undergo atresia and die.

Atresia can be initiated at any stage during the follicular development, but mostly it occurs at the transitional stages between preantral follicle growth and the onset of antrum formation [3, 4]. Granulosa cells degenerate first, while theca cells die at the later stages of development [5]. However, susceptibility to apoptosis and chances of follicle survival depend on the stages of follicle development and the type of survival factor [2]. Cell-cell communication between theca and granulosa cells is mediated by paracrine factors secreted by these cells, essential for oocyte development and regulation of follicular atresia [6–9]. The mesenchymal- epithelial interactions are in part mediated by keratinocyte growth factor (KGF), hepatocyte growth factor (HGF), basic fibroblast growth factor (bFGF), and kit ligand (KITLG) [10, 11].

Theca cell-derived factor, KGF enhance the proliferation of granulosa cells and promotes the preantral follicle growth alone or coupled with FSH by increasing Inhibin-α expression in bovine and rat antral follicles [1, 12]. KGF in a dose-dependent manner suppresses apoptosis by regulating the DNA fragmentation in rat preovulatory and preantral follicles [1]. The granulosa cell-derived factor; KITLG is essential for oocyte development and preantral follicle survival in mouse and human, also KITLG upregulates the HGF expression thereby supporting the proliferation of granulosa cells [13, 14]. KITLG has been reported to inhibit the apoptosis in mice oocytes by modulating the protein/gene expression of Bcl-2 family members [15, 16].

Atresia is regulated by a balance between survival and apoptotic factors [17, 18]. Despite the well-established importance of follicular atresia in ovarian development, the molecular mechanisms underlying the process still remain unclear [19]. Several factors are involved in the regulation of follicular atresia including caspases, death receptor-ligand system, Bcl-2 family, PI3K/Akt signaling, cytokines, and growth factors [20, 21]. In granulosa cells, apoptotic signaling can be initiated by an extrinsic or intrinsic pathway leading to follicular atresia [21, 22]. The Fas-FasL system is the most essential apoptosis extrinsic signaling pathways in granulosa cells. In many species, both Fas and FasL are expressed in granulosa cells and have been suggested to be significant for the process of follicular atresia [23–25]. The expression of both Fas and FasL were upregulated in granulosa cells of atretic follicles than those in healthy follicles in bovine ovaries [25]. In mice, the Fas-activating antibody promotes apoptosis in granulosa cells and initiates atresia [26]. Taken together, these expression patterns indicate the role (or involvement) of FasL-Fas signaling in follicular atresia. In the extrinsic apoptotic pathway, the intracellular death domain (DD) of the Fas interacts with Fas-associated death domain protein (FADD). Further, FADD activates an initiator caspase (procaspase 8) upon interaction through their death-effector domain (DED), leading to receptor aggregation and formation of a death-inducing signaling complex (DISC) [27].

The Fas-FasL system in granulosa cells can be blocked by cFLIP (cellular FLICE-inhibitory protein), a homologue to procaspase-8. cFLIP blocks the Fas-FasL inducible signaling by competing with procaspase-8 and interfering with its binding to FADD. Thus, by inhibiting the DISC formation, cFLIP induces proliferation of granulosa cells and promotes oocyte development [27]. Previous studies on porcine ovaries demonstrated that granulosa cells of healthy follicles had higher expressions of cFLIP mRNA and protein, as compared to the atretic follicles [28, 29].

In bovine follicles, granulosa cell apoptosis is an early feature of atresia and progresses after antrum formation [30]. The average number of primordial follicles in buffalo ovaries ranges from 10,000 to 19,000 which is much lower than those in cattle (∼ 150,000). This lower number of primordial follicles in buffaloes lead to less antral follicles formation and causes more atretic follicles (∼ 92–95 *vs*. 70% for buffalo and cattle, respectively). Interestingly, the primary and secondary follicles are more atretic (> 90%) and the percentage of atresia tertiary follicles ranges from 40 to 75% [31–33]. The accelerated rate of follicular atresia in buffaloes is one of the potential reasons for its poor reproduction [34]. Apoptotic events in granulosa cells and higher follicular atresia in buffalo as compared to that in cattle remains elusive. Though it is likely that this difference between the two species could relate to different rates of germ cell apoptosis but this has still not been addressed.

Despite several reports on the action of pro-survival and pro-apoptotic factors, the literature is still silent on the direct interactions of KGF and KITLG in growth and survival of granulosa cells (GCs). Also, there is lack of substantial evidence suggesting the roles of KGF and KITLG in modulation of Fas-FasL and Bcl-2 pathways during buffalo granulosa proliferation, survival, and oocyte growth [1, 16, 35, 36]. Therefore, a deeper understanding of the underlying intracellular mechanism and factors regulating the process of follicular atresia is critical.

The present study is an attempt to uncover the roles of KGF and KITLG in regulating granulosa cell apoptosis to optimize the reproductive efficiency of buffalo. To address this, the effect of different independent doses (0, 10, 20, and 50 ng/ml) or in combination of KGF and KITLG have been tested to inspect apoptosis in buffalo granulosa cells. Expression studies for both pro-apoptotic (*Fas FasL and Bax*) and anti-apoptotic (*cFLIP, Bcl-2,* and *Bcl-xL*) genes at transcriptional levels were assessed to ascertain the roles of KGF and KITLG in regulating the expression of these factors. In the process, granulosa cell proliferation and differentiation markers (*Inhibin-α* and *bFGF*) were also analyzed in buffalo ovaries.

## Methods

### Reagents and antibodies

All the reagents required for the granulosa cells culture were purchased from Sigma-Aldrich Inc. (St. Louis, MO, USA) unless stated otherwise. The recombinant human KGF and KITLG were obtained from Gibco® (Life Technologies, CA, USA). All the antibodies were obtained from Santa Cruz Biotechnology and Cell Signaling Technology, USA.

### Collection of buffalo ovaries

Buffalo ovaries were obtained from Ghazipur slaughterhouse at New Delhi, India, in accordance with the guidelines of the Institute’s Ethical and Bio-safety committees. The ovaries were placed in chilled normal saline (0.9% NaCl) supplemented with penicillin (100 U/ml) and streptomycin (100 μg/ml) and shipped to the laboratory within 2–3 h. The ovaries were washed in 70% ethanol, followed by treatment in saline solution and disinfected once in 70% ethanol for 30 s, washed again with saline, and then processed immediately. Healthy developing follicles were assessed by the presence of vascularized theca externa and clear amber follicular fluid with no debris.

### Isolation of buffalo granulosa cells (GCs)

For granulosa cell culture, small and medium antral follicles (2–8 mm) were selected for aspiration. The follicular fluid was collected in the sterile and cold condition in a 15 ml centrifuge tube. Chilled PBS having penicillin (100 U/ml), streptomycin (100 μg/ml), and amphotericin B (1.25 μg/ml) were used for follicular fluid collection. Centrifugation at 15,000 rpm for 5–6 min was sufficient for pelleting the granulosa cells from the fluid. The trypan blue and Haemocytometer were used for checking the viability and counting the cells, respectively.

### Culture and treatment of granulosa cells

Viable granulosa cells approximately at the density of 2 × 10^5^ cells per ml were plated in 24-well culture plates (Nunc, Roskilde, Denmark) and Dulbecco’s modified eagle media (DMEM) was used for culturing. Basal DMEM was supplemented with l-glutamine (3 mM), protease-free bovine serum albumin (1 mg/ml), sodium selenite (4 ng/ml), transferrin (2.5 μg/ml), rostenedione (2 μM), bovine insulin (10 ng/ml), non-essential amino acid mix (1.1 mM), ovine FSH (1 ng/ml), human rIGF-1 (1 ng/ml), and with antibiotics penicillin (100 U/ml) and streptomycin (100 μg/ml). Then, cells were then incubated at 37 °C in 5% CO_2_, 95% air for 48 h [37].

### Treatment with recombinant KFG and KITLG

After the initial 48 h of culture, cells were either left untreated (control) or treated with recombinant KGF and/or KITLG proteins. For the experimental conditions, granulosa cells were cultured in different doses of KGF (K) or Kit ligand (KL) independently or (0, 10, 20, and 50 ng/ml) in combination (K + KL). After 48 h of treatment, media was carefully removed and cells were lysed and stored at − 80 °C or processed for RNA isolation followed by gene expression by real-time PCR.

### RNA isolation and cDNA synthesis

Total RNAs were extracted from both control and treated buffalo granulosa cells using TRIzol® Reagent (Invitrogen, Life Technologies). The RNA concentration and purity was determined using a spectrophotometer (NanoDrop ND-1000, Thermo Fisher Scientific, USA). The integrity of RNAs was tested on 1% formaldehyde agarose gel and the presence of genomic DNA were checked with PCR using *β-actin* primers (Frd: 5′CAGATCATGTTCGAGACCTTCAA3′ and Rev: 5′GATGATCTTGATCTTCATTGTGCTG3′). Following the total RNA isolation from control and treated GCs, ~ 1.0 μg of RNAs were taken for cDNA synthesis using Verso cDNA synthesis kit (Thermo Scientific, USA) for each reaction. Reactions were prepared as per the manufacture’s protocol and incubated first at 42 °C for 45 min followed by 95 °C for 2 min and finally at 4 °C. For the confirmation of synthesis, an aliquot of the synthesized cDNA was subjected to PCR using *β-actin* primers.

### Quantitative expression of anti-/pro-apoptotic genes

The mRNA expression of anti-/pro-apoptotic genes in both control and treated granulosa cells were analyzed using real-time PCR. The reaction was performed employing Power SYBR® green (Part no. 4367659; ABI, Warrington, UK) on Sequence Detection System 7500 (Applied Biosystems, USA). The 18S ribosomal RNA gene was taken as the internal control for each reaction. The expression level of each candidate gene was quantified on the basis of *C*_t_ value (delta-delta *C*_t_ method). Primers for assessing the relative expression for each gene were designed on Primer Express*®* Software v3.0, given in Table 1. The real-time PCR reactions were set up in triplicates employing universal cycling conditions recommended by ABI (50 °C for 2 min, 95 °C for 10 min and 40 cycles for 95 °C for 15 s and 60 °C for 1 min). To adjust for variations in the input sample, the average *C*_t_ values for the individual target genes were normalized against the average *C*_t_ values for the internal control (18S) [ΔC_t(Target)_ = [*C*_t_ (Target) – C_t_(18S)] [38, 39].

**Table 1:** Oligonucleotide primer sequences and amplicon size used for real-time PCR

Gene	Primer sequence 5′-3′	Size (bp)	Accession number
*18S*	Frd-AGAAACGGCTACCACATCCAA	140	AF176811.1
	Rev-CGGAAGGATTTAAAGTGGACTCA		
*Fas*	Frd-CTGTTTCTGGACCATTGT	92	NM_174662.2
	Rev-GGAGTTCGCTTCAGTAAT		
*FasL*	Frd-TGGAATGGGAAGACACCTATGG	120	NM_001098859.2
	Rev-GACCCCGGAAGTACACTTTGG		
*Inhibin α*	Frd-CTGAGCCCGAGGACCAAGA	95	NM_174094.4
	Rev-CTCCTCAGCCTCTCCAGCAT		
*bFGF2*	Frd-TTTCTTTTTTGAACGATTGGAGTCT	85	NM_174056.4
	Rev-TCGTTTCAGTGCCACATACCA		
*c-kit*	Frd-CAGAAATCCTGACGCATGACA	105	DQ314491.1
	Rev-GCTCGGTTCCTGGACAAAAG		
*FGFR2*	Frd-CGGCCCTCCTTCAATTTAGTT	150	NM_001205310.1
	Rev-ACTGATCATGGCGGCATCTC		
*cFLIP*	Frd-AGTCATCCATCAGGTAGAAGAAG	77	NM_001012281.1
	Rev-AGCAACATCTCGGCACAA		
*Bax*	Frd-AAGAAGCTGAGCGAGTGT	78	NM_173894.1
	Rev-AGCTGCGATCATCCTCTG		
*Bcl-2*	Frd-ACCTGCACACCTGGATCCA	100	NM_001166486.1
	Rev-AGAGACAGCCAGGAGAAATCAAA		
*Bcl-xL*	Frd-AAGCGTAGACAAGGAGAT	76	NM_001077486.2
	Rev-TAGGTGGTCATTCAGGTAA		

*18S* 18S ribosomal RNA gene, *Fas* Fas cell surface death receptor, *FasL* TNF superfamily, member 6, *cFLIP* Cellular FLICE-like inhibitory protein, *Inhibin α* Inhibin alpha, *bFGF2* Basic Fibroblast growth factor 2, *C-kit* C-kit receptor, *FGFR2* Fibroblast growth factor receptor 2, *Bcl-xL* B cell lymphoma-extra-large, *Bcl2* B cell CLL/lymphoma 2, *Bax* BCL2-associated X protein

### Data interpretation: statistical analysis

The Real TimePCR data of all the genes quantified was subjected to student t-test to determine the significance employing MS Excel. All the results are presented as the mean ± SEM of three independent experiments and values (*P* < 0.05) are statistically significant.

### Immunohistochemistry

KGF and KITLG were immunolocalized in buffalo ovary tissue sections using anti-KGF/KITLG antibodies (Santa Cruz Biotechnology, USA). Paraffin blocks were prepared by immersing tissue samples in neutral buffer containing 10% formalin for 8 h. Tissues were dehydrated in ascending grade of ethanol, infiltrated and embedded in low melting paraffin at 56 °C in a heated oven. The tissue paraffin mold was solidified on a cold plate to form a block. Fixed tissues were sectioned (5 μM) using a microtome (MRS3500 Histoline Laboratories, Italy). Tissue sections were deparaffinized and rehydrated in descending grades of ethanol (100–50%). Endogenous tissue peroxidase was blocked by incubating the sections with 3% hydrogen peroxide solution in methanol for 10 min. Sections were treated with 1% Triton X-100 followed by blocking with 1% bovine serum albumin (BSA). Specific antibodies corresponding to KGF and KITLG (1:100) were added to each tissue section and incubated overnight at 4 °C in a humidified chamber. The appropriate HRP conjugated secondary antibody (1:1000) and diaminobenzidine (DAB) chromogen and H_2_O_2_ as a substrate were used to detect the binding of primary antibodies. Sections were counterstained with Harris hematoxylin stain, dehydrated with a serial gradient of ethanol (50–100%), air-dried, and mounted in DPX (mounting media). The screening was done under differential interference contrast (DIC) Nikon Eclipse T2 Microscope.

## Results

### KGF and KITLG downregulates Fas/FasL by and increasing cFLIP expression

**Fig. 1:**
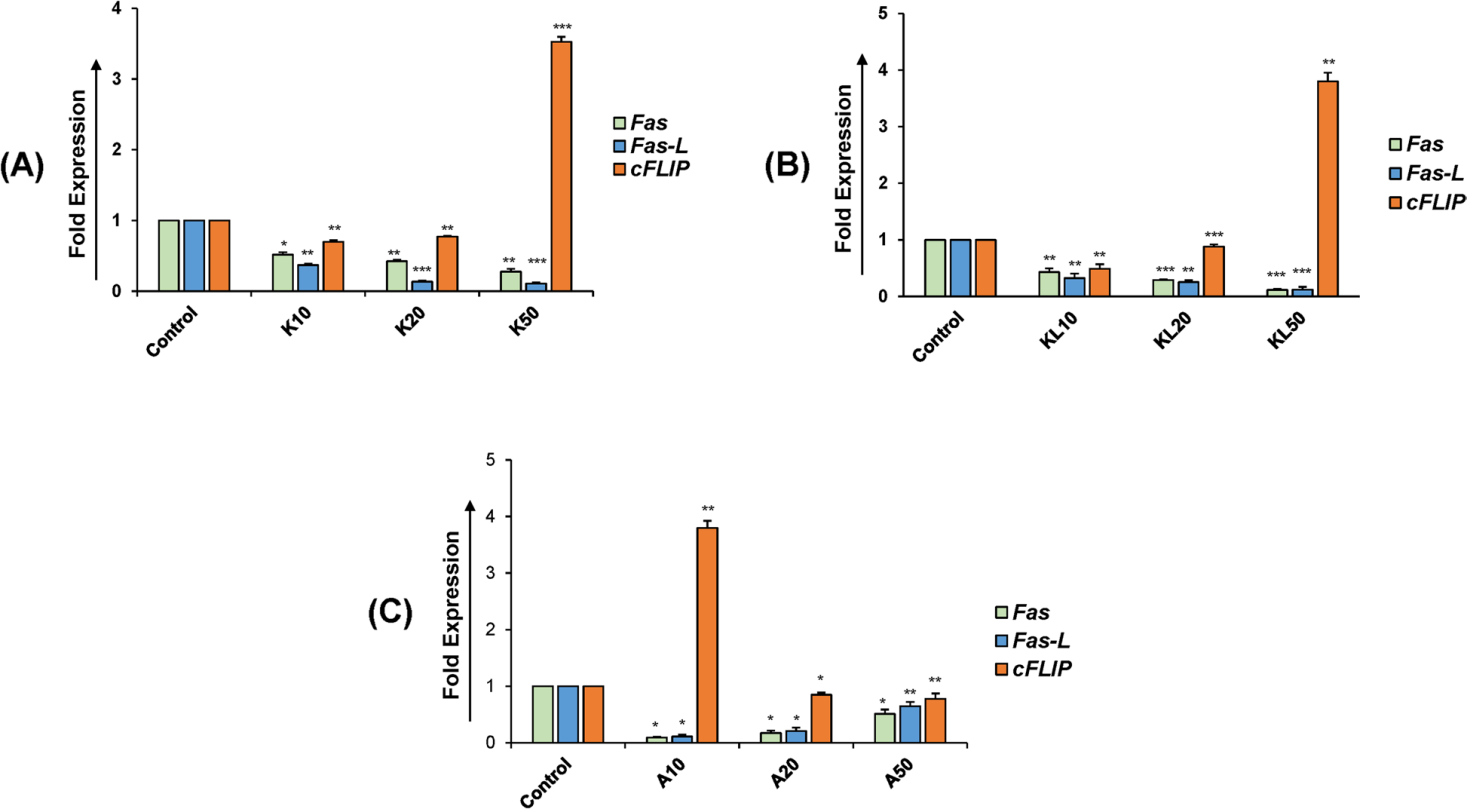
The relative expression of *Fas* and *FasL* mRNA in KGF and KITLG treated granulosa cells. Expression of Fas and FasL were significantly down regulated in a dose-dependent manner by KGF and KITLG treatment and was found to be lowest at the concentration of 50 ng/ml (**A**, **B**). Expression of *cFLIP* was significantly increased and was in direct proportion to the dosage of KGF and KITLG and was found to be maximum at the concentration of 50 ng/ml. When granulosa cells were treated with both KGF and KITLG, concentration of 10 ng/ml was found to be more significant in regulation of Fas-FasL pathway by *cFLIP* (**C**). Data are presented as relative expression of *Fas, FasL*, *and cFLIP* mRNA/18S mRNA normalized to untreated control values. K represents the KGF, KITLG is denoted by KL and A is KGF+KITLG. The dose concentrations are defined as 10, 20, and 50 ng/ml for both KGF and KITLG. Values are expressed as the mean ± SD of three experiments; *P < 0.05, **P < 0.01, ***P < 0.001 vs. the control (non-treated)

The real-time PCR analysis showed a marked decrease in mRNA expressions of *Fas* and *FasL* in granulosa cells with all the different concentrations (0, 10, and 20 ng/ml) of KGF and KITLG (Fig. 1A, B). Significant downregulation of *Fas* and *FasL* was observed at 50 ng/ml (*P* < 0.01) (Fig. 1A, B). On the other hand, mRNA expression of *cFLIP* was sharply increased in KGF- and KITLG-treated granulosa cells and upregulation was most intense (*P* < 0.001) at 50 ng/ml (Fig. 1A, B). In contrast, when KGF and KITLG were used together, downregulation of *Fas*/*FasL* and upregulation of *cFLIP* was observed at 10 ng/ml (Fig. 1C). These observations suggest that the KGF and KITLG prevent the apoptosis of granulosa cells by upregulating cFLIP and inhibiting the Fas/FasL pathway. Our results provide an insight into the quintessential cross-talk between Fas/FasL apoptosis pathway and cFLIP-mediated anti-apoptotic regulation in buffalo ovarian follicle atresia.

### Regulation of Bcl-2 family members

**Fig. 2:**
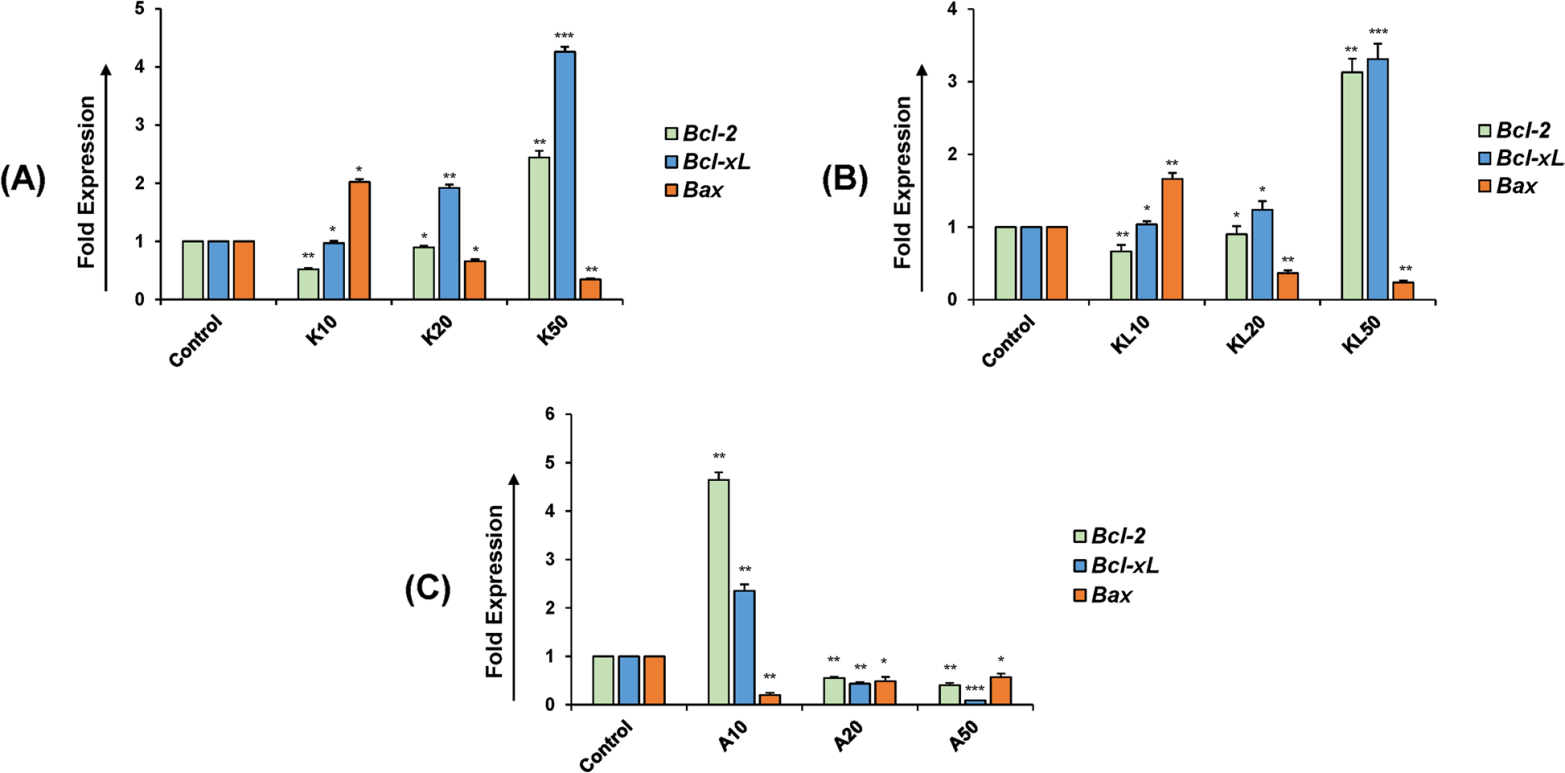
*Bcl-2*, *Bcl-xL* and *BAX* genes expression in granulosa cells with KGF and KITLG. **A**, **B** A significant increase in *Bcl-2* and *Bcl-xL* expression was observed and Bax was down-regulated in bovine granulosa cells with KGF and KITLG treatment. **C** Treatment of granulosa cells with combination of KGF and KITLG increased the expression levels of anti-apoptotic proteins (*Bcl-2* and *Bcl-xL*) and decreased significantly (**P < 0.01) the pro-apoptotic protein *Bax* at 10 ng/ml. K represents the KGF, KITLG is denoted by KL and A is KGF+KITLG. The dose concentration are defined as 10, 20, and 50 ng/ml for both KGF and KITLG. Values are expressed as the mean ± SD of three experiments; *P < 0.05, **P < 0.01, ***P < 0.001 vs. the control (non-treated)

The data showed the significantly increased expression of *Bcl-2* and *Bcl-xL* in treated granulosa cells, while the expression of *Bax* was decreased in a dose-dependent manner (Fig. 2A, B). The expression of *Bcl-2* and *Bcl-xL* was further analyzed in the presence of both KGF and KITLG, which showed the highest expression at 10 ng/ml. The expression of *Bax* with 10 ng/ml dose of KGF+KITLG is shown in Fig. 2C. These observations indicate that KGF and KITLG carry out their anti-apoptotic function in buffalo granulosa cells by regulating the expression of Bcl-2 and Bcl-xL (anti-apoptotic) and Bax (pro-apoptotic) genes.

### KGF and KITLG promoted granulosa cell growth

**Fig. 3:**
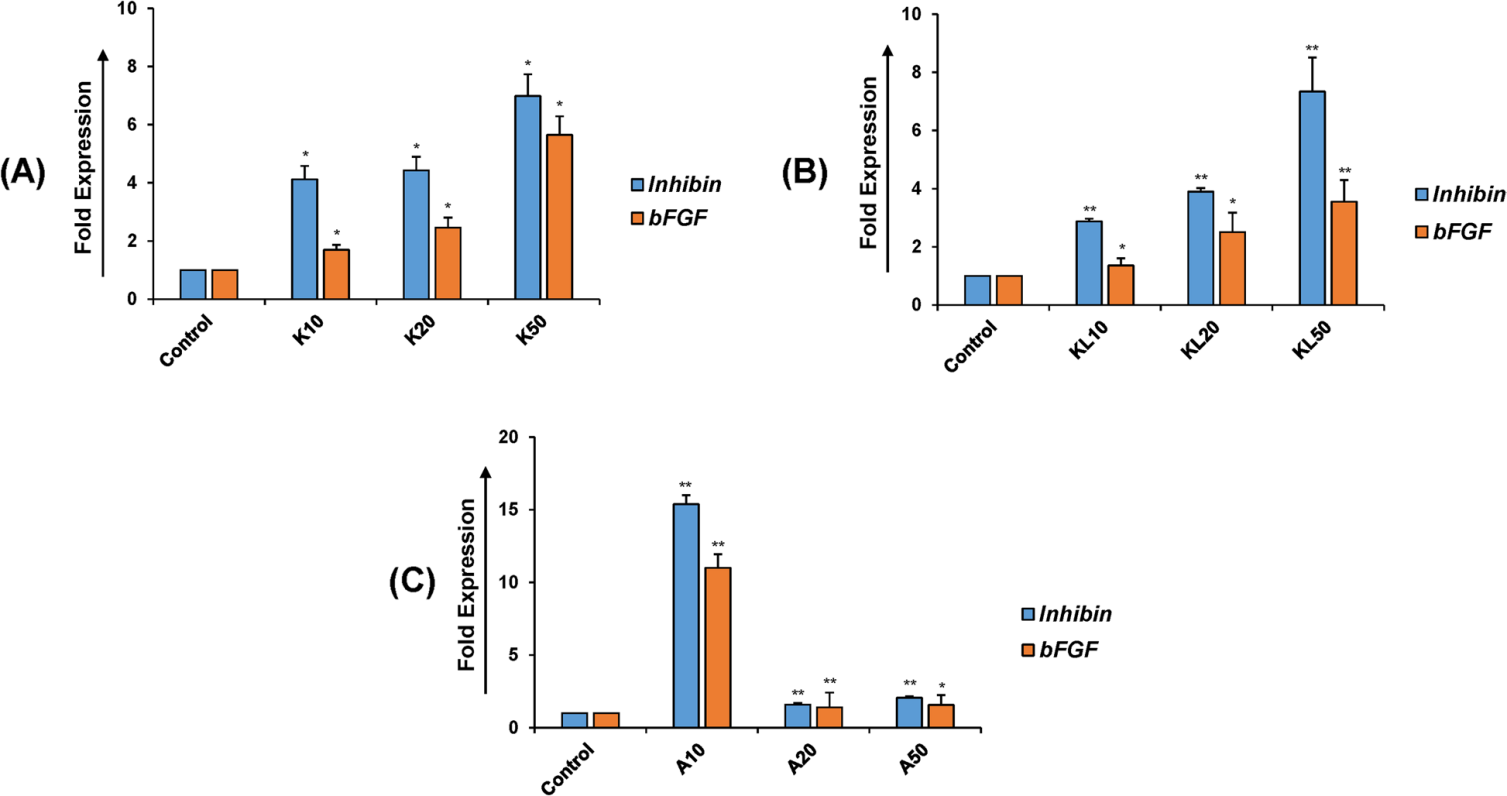
Treatment with KGF and KITLG enhances the growth of granulosa cells. The mRNA expressions in cultured granulosa cells in response to KGF and KITLG treatment were determined using Real-time PCR. *Inhibin-α* was consistently increased with doses dependent manner of KFG (**A**) and KITLG (**B**). The mRNA expression of *bFGF* remained almost unchanged at 10 and 20 ng/ml as compared to the control with both KGF (**A**) and KITLG (**B**) treatment and significantly upregulated at 50 ng/ml concentration. Upregulation for *Inhibin*-*α* and *bFGF* expression was more consistent and significant at the dose of 50 ng/ml as compared to that of 10 and 20 ng/ml. **C** Combined treatment of KGF and KITLG at 10 ng/ml resulted in increased expression of *Inhibin*-*α* and *bFGF* (> 10-fold), whereas there is no change at the dosage of 10 and 20 ng/ml. These observations suggest the role of KGF and KITLG in growth of granulosa cell. K represents the KGF, KITLG is denoted by KL and A is KGF + KITLG. The dose concentrations are defined as 10, 20, and 50 ng/ml for both KGF and KITLG. Values are expressed as the mean ± SD of three experiments; *P < 0.05, **P < 0.01, ***P < 0.001 vs. the control (non-treated)

The expression of *Inhibin*-*α* was found to be consistently upregulated with an increase in the doses of KGF and KITLG in granulosa cells (Fig. 3A, B). Also, the expression of the *bFGF* gene was upregulated in the treated granulosa cells at the dosage of 20 and 50 ng/ml, respectively (Fig. 3A, B). Clearly, upregulation in expression for *Inhibin*-*α* and *bFGF* was more consistent and was found to be significant at the dose of 50 ng/ml as compared to that of 10 and 20 ng/ml. Similarly, strong enhancement (> 10-fold increase) in the expression of *Inhibin*-*α* and *bFGF* was observed when granulosa cells were treated with both KGF and KITLG proteins (Fig. 3C).

### Synergistic autoregulation of KGF and KITLG

**Fig. 4:**
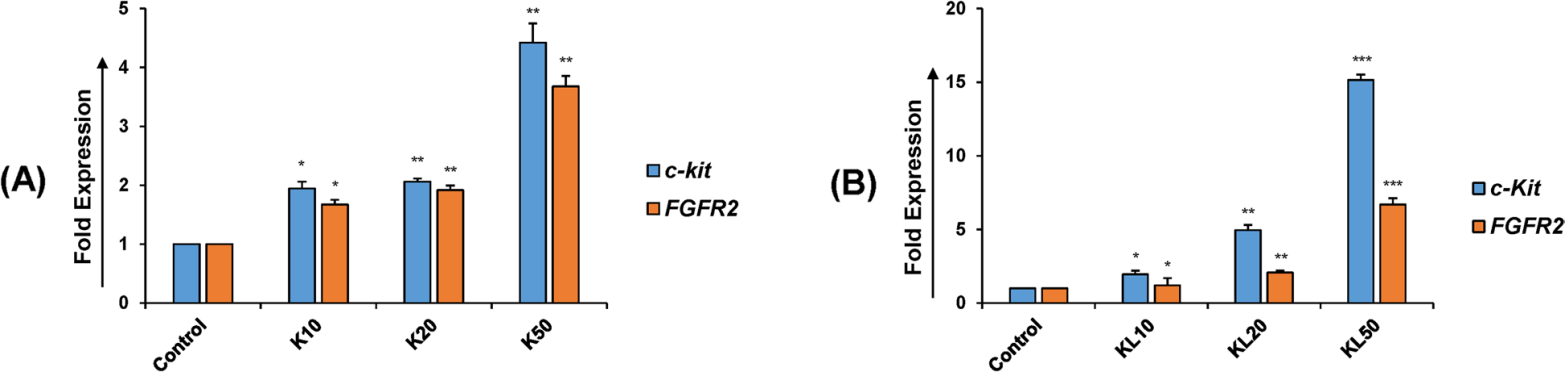
Autocrine action of KGF and KITLG in buffalo granulosa cells. **A** A significant increase was observed for *c-kit* and *FGFR2* expression in a dose-dependent manner of KGF treated cells. **B** The KITLG treated cells resulted in upregulation of *c-kit* (by > 10 folds) and *FGFR2* (by > 5 folds) (**B**). These observations suggest that KGF and KITLG act synergistically by regulating each other’s expression across their site of production and action. K represents the KGF, KITLG is denoted by KL. The dose concentrations are defined as 10, 20, and 50 ng/ml for both KGF and KITLG. Values are expressed as the mean ± SD of three experiments; *P < 0.05, **P < 0.01, ***P < 0.001 vs. the control (non-treated)

**Fig. 5:**
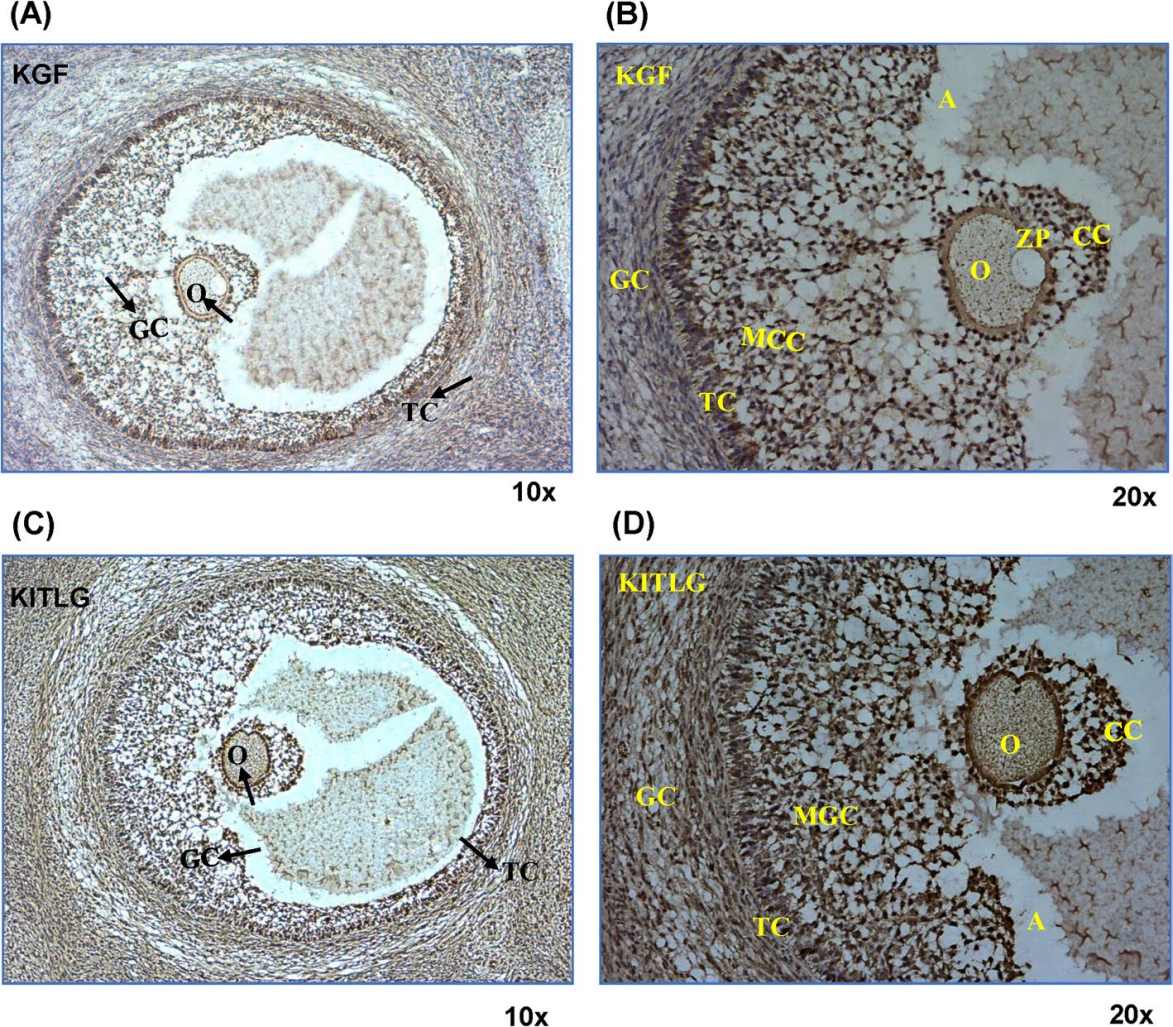
Immunohistochemical localization of KGF and KITLG in buffalo ovarian sections. Brown color represents staining of targeted proteins, KGF and KITLG, while blue color is background counter staining. Arrow heads indicate the oocyte, granulosa and theca cells. **A**, **B** Expression of KGF at granulosa and theca cells. **C**, **D** show the expression of KITLG at all three sites at granulosa, theca and oocyte. Magnification is × 10 and × 20. O: Oocyte, ZP: Zona pellucida, GC: Granulosa cells, A: Antrum, TC: Theca cells, MGC: Mural GC, CC: cumulus cells

Interaction between KGF and KITLG and common pathways of their anti-apoptotic functions are possible owing to the positive feedback loop between the two (Panwar et al. 2015). To address this hypothesis, the expression of the KGF receptor (*FGFR2*) and KITLG receptor (*c-kit)* was analyzed by real-time PCR. Results showed that the expression of the KGF receptor (*FGFR2*) was markedly upregulated in KGF and KITLG treated granulosa cells in a dose dependent manner as compared to control granulosa cells (Fig. 4A, B). These data suggest that KGF and KITLG not only regulate their own expression but also the expression of each other’s receptors involving feedback loop mechanism. Additionally, employing IHC, the KGF, and KITLG proteins were localized in granulosa and theca cells of secondary follicles in the buffalo ovarian sections, respectively (Fig. 5A, B). A positive immunoreaction for KITLG was also observed in the oocyte (Fig. 5C, D), but not for KGF. Thus, localization site of expression and action indicated the synergizing effect of growth factor-mediated granulosa-theca cell interactions.

## Discussion

This present study on *Bubalus bubalis* in an attempt to extend the work on a significant livestock resource in India and several countries of South Asia and the Mediterranean regions. The buffaloes have poor reproductive performance, therefore, assisted reproduction may increase the success of buffalo breeding. As more than 99% of all follicles undergo atresia and die, quality of oocytes can be improved by regulating the granulosa cell apoptosis with ovarian factors. In this view, we have explored the potential role of two important growth factors, KGF and KITLG by determining their protein expression and immunolocalization in regulation of granulosa cell apoptosis.

Several signaling pathways and ovarian factors are involved in the regulation of follicular atresia. It is a complex process regulated by the fine-tuning of pro-survival and pro-apoptotic factors. Granulosa cell apoptosis plays a critical role in the growth and development of oocytes. The role of KGF and KITLG as significant factors in regulating follicular survival and growth has been reported in many species [1, 11, 35, 36]. However, the effect of KGF and KITLG on apoptosis of granulosa cells in buffalo has not been explored. We analyzed apoptosis in buffalo granulosa cells treated with different concentrations of recombinant KGF and KITLG proteins. Apoptosis of granulosa cells is predominantly mediated by cell death-ligand/receptor system and intrinsic pathway controlled by Bcl-2 family members [26, 40, 41]. The balance between pro-apoptotic and anti-apoptotic factors of Bcl-2 family members is directly related to follicular development and atresia [42]. The relative expression studies showed the upregulation of *Bcl-2* and *Bcl-xL* and downregulation of *Bax* in rKGF and rKITLG treated granulosa cells. Reports are available on the anti-apoptotic effect of KFG and KITLG in mice oocytes [1, 16]. Our results demonstrate their anti-apoptotic activity in granulosa cell as well. The Fas-FasL system-mediated apoptosis constitutes a major pathway involved in the initiation of apoptosis in granulosa cells [23]. Lower *FasL* mRNA expression was observed upon treatment with KFG and KITLG, compared to the untreated buffalo granulosa cells. The cFLIP is an intracellular anti-apoptotic protein, a homolog of procaspase-8 (also called FLICE) [29, 41, 43, 44]. Granulosa cells produce KITLG that bind to its tyrosine kinase receptor, c-kit, localized on the theca cells and oocyte to stimulate follicle recruitment and survival [45, 46]. cFLIP mRNA and protein is reported to be highly expressed in granulosa cells of healthy follicles in pig and downregulated during atresia [29, 41, 43, 44]. In our study, the increased expression of *cFLIP* mRNA in granulosa cells treated with 50 ng/ml of rKGF and rKITLG suggested that both these factors inhibit the Fas-FasL signaling pathway by modulation of *cFLIP* expression.

Earlier studies have shown that KGF stimulated the proliferation of bovine granulosa cells and promoted the growth of rat preantral follicles by increasing Inhibin-α protein content [1, 47]. The bFGF has also been shown to be important in promoting the primordial to primary follicle transition in the rat. It is localized in granulosa cell of developing preantral follicles of rat and cow [48, 49]. Bovine granulosa cells have been shown to produce bFGF in the preantral and antral follicle stages, important for granulosa cell mitosis [50]. Our results suggest that KGF and KITLG not only inhibit granulosa cell apoptosis but also promote granulosa cell proliferation by upregulating the expression of *Inhibin-α* and *bFGF* genes.

The interaction between KGF and KITLG proteins seems imperative, but their mechanism of action in the regulation of folliculogenesis is still unclear [36, 51]. Although in mammals, the interaction of KGF and KITLG is considered to be critical for the proliferation and growth of follicular cells and oocytes during different stages, the biological requirements for this event vary across the species [51]. Previous studies have shown that KGF and KITLG regulate each other at the transcriptional level, however, information on their interaction was lacking [35, 36, 52]. Our previous study has shown the successful in vivo interaction between buffalo KGF and KITLG proteins, followed by the prediction of their binding interface [52]. Protein expression and immunolocalization of both KGF and KITLG was detected in antral follicles using IHC. KITLG is expressed by the granulosa cells and mediates its action through its receptor present on the theca and oocyte. Our IHC observations confirmed their expression in theca and oocyte. Similarly, KGF is localized in the theca cells and granulosa cells. Thus, IHC findings suggest the autocrine/paracrine interplay of KGF and KITLG protein in buffalo ovary, as both the proteins were localized at their site of production and site of action as well.

## Conclusions

The oocytes development is a crucial step in folliculogenesis to induce fertilization. Our study suggests the potential role of KGF and KITLG in preventing and regulating granulosa cell apoptosis. Thus, both KGF and KITLG can be used as growth factors in buffalo oocytes cultures for maturation of oocytes, in vitro maturation (IVM) and in vitro fertilization (IVF) procedures.

## Data Availability

The data used to support the findings of the present study are included within the article
